# Effect of *BRCA2 *sequence variants predicted to disrupt exonic splice enhancers on *BRCA2 *transcripts

**DOI:** 10.1186/1471-2350-11-80

**Published:** 2010-05-28

**Authors:** Phillip J Whiley, Christopher A Pettigrew, Brooke L Brewster, Logan C Walker, Amanda B Spurdle, Melissa A Brown

**Affiliations:** 1 School of Chemistry and Molecular Biosciences, The University of Queensland, Australia; 2 Genetics and Population Health Division, Queensland Institute of Medical Research, Herston Queensland, Australia; 3 Peter MacCallum Cancer Centre, Melbourne, Australia; 4 Current Address: Tumour Biology Laboratory, Department of Biochemistry, University College Cork, Cork, Ireland

## Abstract

**Background:**

Genetic screening of breast cancer patients and their families have identified a number of variants of unknown clinical significance in the breast cancer susceptibility genes, *BRCA1 *and *BRCA2*. Evaluation of such unclassified variants may be assisted by web-based bioinformatic prediction tools, although accurate prediction of aberrant splicing by unclassified variants affecting exonic splice enhancers (ESEs) remains a challenge.

**Methods:**

This study used a combination of RT-PCR analysis and splicing reporter minigene assays to assess five unclassified variants in the *BRCA2 *gene that we had previously predicted to disrupt an ESE using bioinformatic approaches.

**Results:**

Analysis of *BRCA2 *c.8308 G > A (p.Ala2770Thr) by mRNA analysis, and *BRCA2 *c.8962A > G (p.Ser2988Gly), *BRCA2 *c.8972G > A (p.Arg2991His), *BRCA2 *c.9172A > G (p.Ser3058Gly), and *BRCA2 *c.9213G > T (p.Glu3071Asp) by a minigene assay, revealed no evidence for aberrant splicing.

**Conclusions:**

These results illustrate the need for improved methods for predicting functional ESEs and the potential consequences of sequence variants contained therein.

## Background

DNA sequence variants of unknown clinical significance are regularly identified when individuals with a family history of breast cancer are screened for mutations in the *BRCA1 *and *BRCA2 *genes. Determining the clinical relevance of these unclassified variants, particularly rare exonic unclassified variants, is challenging. Currently, functional assays designed to assess the pathogenicity of exonic unclassified variants usually aim to determine the effect on protein function, and do not take into account the potential effect the UV may have on RNA function. Defects in RNA function, including defects in RNA splicing, stability and translation, are likely to underly the pathogenicity of a significant proportion of unclassified variants (reviewed in [1]). For example, sequence variants in exonic splice enhancers (ESEs) that result in either abnormal splicing or induce the skipping and therefore rescue of deleterious non-sense mutations, have previously been reported in multiple disease-associated genes, including *BRCA1 *and *BRCA2 *[2-6],

Whilst predicting the consequences of unclassified variants in the consensus donor and acceptor dinucleotide sites flanking exons can be done with reasonable confidence, forecasting the effect of exonic unclassified variants mapping to ESEs is much more difficult. This is in part due to fact that ESEs are relatively poorly defined and the SR proteins involved in recognition of ESEs may recognize a wide variety of sequences [7]. Several approaches have been proposed to assist in the identification of *bona fide *active ESEs, including evolutionary conservation, distance from the intron-exon boundaries, and the application of more stringent thresholds when using bioinformatic prediction tools [4,8,9].

In a previous study [10], our group used the ESE prediction tool ESEfinder to identify total of 1,114 ESEs across the *BRCA2 *transcript. The total number of predicted ESEs was reduced to 31 by introducing custom thresholds for four SR proteins involved in enhancer activity, restricting the length of exonic sequence to the first and final 125 base pairs (bp), and assessing ESEs for sequence conservation. Significantly, twenty of these prioritized ESEs colocalized with unclassified variants reported to the Breast Cancer Information Core (BIC, http://research.nhgri.nih.gov/bic/) and the Kathleen Cuningham Foundation Consortium for research into Familial Breast cancer (kConFab, http://www.kconfab.org). In the conclusions of our previous paper [10], we recommended that the unclassified variants prioritised in the study be experimentally analysed for splicing disruption to verify the *in silico *predictions.

In this study we analysed five *BRCA2 *unclassified variants that we had previously predicted to either cause loss of an ESE motif (denoted using HGVS nomenclature ([11]) as: *BRCA2 *c.8962A > G (p.Ser2988Gly), *BRCA2 *c.8972G > A (p.Arg2991His) and *BRCA2 *c.9213G > T (p.Glu3071Asp)) or to decrease the score of the predicted ESE motif (*BRCA2 *c.9172A > G (p.Ser3058Gly) and *BRCA2 *c.8308 G > A (p.Ala2770Thr)) [10]. A source of RNA was only available for one of these variants (*BRCA2 *c.8308 G > A (p.Ala2770Thr)), which was analysed for splicing defects by RT-PCR. For the remaining four, we used a minigene approach [12-14] to examine the effect of each variant on splicing.

## Methods

### LCL mRNA analysis of *BRCA2 *c.8308 G > A (p.Ala2770Thr)

A lymphoblastoid cell line (LCL) generated from the lymphocytes of a patient carrying *BRCA2 *c.8308 G > A (p.Ala2770Thr) was obtained from kConFab for mRNA analysis. RNA was extracted from the LCL as well as two independent normal control LCLs after treatment with and without cyclohexamide, utilised to reduce the incidence of nonsense mediated decay (NMD). *BRCA2 *c.8308 G > A (p.Ala2770Thr) is located in exon 18, and PCR primers were designed to amplify *BRCA2 *transcripts from exons 16 to 19. *BRCA2 *PCRs were performed in 20 μL reactions over 35 cycles with recombinant Taq, using oligo dT primed cDNA and 40 ng of each primer. The primers used were designed to amplify a product spanning exon 18, which harbours the unclassified variant *BRCA2 *c.8308 G > A (p.Ala2770Thr) (Exon 16 for: 5' TGATGGAAAGGCTGGAAAAG-3' and Exon 19 Rev: 5'-GCAGGCCGAGTACTGTTAGC-3').

### Splicing reporter minigene constructs

Minigene constructs containing *BRCA2 *exons 23, intron 23 and exon 24, along with 120 nucleotides of intron 22 and 134 nucleotides of intron 24, were synthesized by Genscript (Genscript Corp, NJ) and cloned into the multiple cloning site of the pSPL3 plasmid (Invitrogen). pSPL3 is an exon trapping vector that contains a splice donor and acceptor site and has been widely used to study the products of pre-mRNA splicing [15]. Minigenes containing the wild type sequence, a positive control, which contains a variant (*BRCA2 *c.9117G > A (p.Pro3039Pro) known to cause skipping of exon 23 [16], or the *BRCA2 *unclassified variants: c.8962A > G (p.Ser2988Gly), c.8972G > A (p.Arg2991His), c.9172A > G (p.Ser3058Gly), and c.9213G > T (p.Glu3071Asp) were generated.

### Cell Culture and transfection

MDA-MB-231 cells were maintained using DMEM (Invitrogen, CA) with 10% foetal bovine serum and 1% penicillin/streptomycin/anti-mycotic. Cells were pre-plated 24 hours prior to transfection in antibiotic free DMEM with 10% foetal bovine serum. Cell cultures were transfected at 90-95% confluence using Lipofectamine 2000 (Invitrogen, CA) according to the manufacturer's instructions, with vectors containing each of the constructs and a vector only control. Cells were then cultured for 48 hours before harvesting for RNA analysis.

### Reverse transcriptase PCR and sequence confirmation

RNA was extracted using Trizol (Invitrogen, CA) and treated with DNase to reduce DNA contamination using DNA-free (Ambion, TX). cDNA was synthesized using 500 ng of DNase treated RNA, using Superscript First Strand Synthesis System III, according to the manufacturer's instructions (Invitrogen, CA). cDNAs corresponding to minigene transcripts were amplified using the primers SD6 (TCTGAGTCACCTGGACAACC) and SA2 (ATCTCAGTGGTATTTGTGAGC) and Amplitaq Gold (Applied Biosystems, Victoria, Australia) under the following conditions: 95°C for 5 mins, 35 cycles of 95°C for 30 secs, 55°C for 30 secs and 72°C for 1 min followed by a final extension step of 72°C for 5 mins. All PCR products were cloned using pGEM^®^-T Easy Vector (Promega Corporation, WI) according to the manufacturer's instructions. Transformant culture (JM109 competent *E-coli*) was spread on LB-ampicillin/IPTG/X-gal plates. Recombinant clones selected by blue-white selection were mixed with 5 μl of water and used as DNA template for PCR under the conditions outlined above, and sequenced using Big-Dye Terminator version 3.1 sequencing chemistry.

## Results

### No evidence of splicing abnormalities caused by *BRCA2 *c.8308 G > A (p.Ala2770Thr)

**Figure 1:**
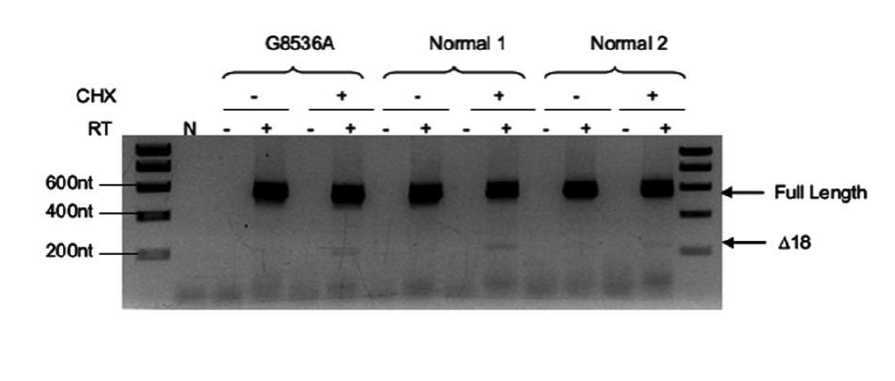
**No evidence for altered splicing of transcripts derived from an LCL generated from a *BRCA2 *c.8308 G > A (p.Ala2770Thr) carrier**. LCLs from a carrier of *BRCA2 *c.8308 G > A (p.Ala2770Thr) or two *BRCA2 *normal LCL controls were analysed by RT-PCR using primers specific for *BRCA2 *exons 16 and 19. CHX indicates cycloheximide treatment, which was used to examine transcript stability, RT indicates reverse transcriptase. N indicates no template control.

To determine the consequences of *BRCA2 *c.8308 G > A (p.Ala2770Thr) on splicing, we performed RT-PCR analysis of a lymphoblastoid cell-line (LCL) generated from the lymphocytes of a patient carrying this UV, and two LCLs from normal controls. PCR products from all samples matched in size to the wild-type *BRCA2 *mRNA and the previously reported *BRCA2*-Δ18 [17] (Figure 1).

### No evidence of splicing abnormalities caused by *BRCA2 *c.8962A > G (p.Ser2988Gly), *BRCA2 *c.8972G > A (p.Arg2991His), *BRCA2 *c.9172A > G (p.Ser3058Gly), or *BRCA2 *c.9213G > T (p.Glu3071Asp)

**Figure 2:**
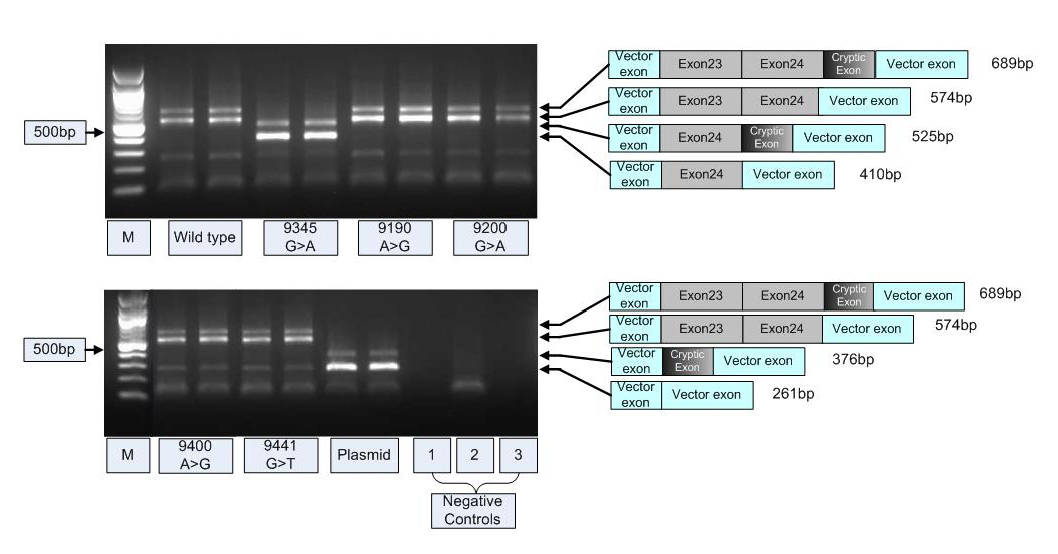
**No evidence for altered splicing of transcripts derived from minigenes incorporating the *BRCA2 *unclassified variants: c.8962A > G (p.Ser2988Gly), c.8972G > A (p.Arg2991His), c.9172A > G (p.Ser3058Gly), and c.9213G > T (p.Glu3071Asp)**. RT-PCR products from duplicate cell culture experiments for each minigene construct and a vector only control. The vector only sample does not contain an insert and therefore produces a 261 bp band made up of fragments of pSPL3 constitutive exons. The positive control, *BRCA2 *c.9117G > A (p.Pro3039Pro) produces a 410 bp band representing the vector exons and *BRCA2 *exon 24. The higher molecular weight band in all samples includes a 115 bp cryptic exon. The 574 bp band seen in all unclassified variants and the wildtype represents the vector exons and *BRCA2 *exons 23 and 24. Negative controls include: 1. No reverse transcriptase in cDNA synthesis of the wild type construct; 2. No template for the PCR; 3. Negative control for transfection. Fragments resolved 1.5% agarose gel stained with ethidium bromide.

To determine the consequences of *BRCA2 *c.8962A > G (p.Ser2988Gly), *BRCA2 *c.8972G > A (p.Arg2991His), *BRCA2 *c.9172A > G (p.Ser3058Gly), and *BRCA2 *c.9213G > T (p.Glu3071Asp) on splicing, minigene constructs containing *BRCA2 *exons 23 and 24 and flanking intron sequences, and either wild-type, positive control or unclassified variant sequences, were introduced into cells and their respective transcripts analysed by RT-PCR and DNA sequencing. As expected, a normal splicing product containing exons 23 and 24 was observed for the wild-type constructs, whereas a smaller product containing exon 24 only was observed upon analysis of the positive control *BRCA2 *c.9117G > A (p.Pro3039Pro), which has been previously reported to induce aberrant splicing (Figure 2). Sequence analysis also revealed that PCR products contained an additional 115 base pair sequence, which corresponded to a cryptic exon derived from intronic sequence downstream from the multiple cloning site within the vector, and which has been reported previously [18]. Analysis of the minigenes containing each of the four tested unclassified variants resulted in PCR products that were indistinguishable from that generated by the wild-type construct (Figure 2). Sequence analysis of the PCR product confirmed the presence of the unclassified variant sequence (data not shown).

## Discussion

This study analysed the pre-mRNA splicing patterns associated with the presence of five *BRCA2 *unclassified variants colocalising with and predicted to disrupt conserved ESEs. Unexpectedly, we found no evidence for altered splicing associated with any of these unclassified variants. For the UV analysed using an LCL, possible explanations for the lack of aberrant splicing include that the effects of the UV are only evident in mammary epithelia and not lymphoblastoid cells, although there are multiple precedents for using LCL RT-PCR analysis to identify pathogenic splicing abnormalities [19]. Possible explanations for the lack of aberrant splicing caused by unclassified variants introduced into our minigene include that the unclassified variants actually affect ESEs that regulate splicing of exons not included in the minigene construct, or that the effect of the UV on the ESE is dependent on a splicing factor that is not expressed or fully functional in the cell-line that was used in these studies. The fact that our positive and negative controls worked in both the minigene experiments and that previous studies have shown concordance between results obtained from minigene assays using cells from a tissue not related to the target [14], suggest that these possibilities are unlikely to explain our results.

A more plausible explanation for our data is that the ESEs predicted bioinformatically are not true ESEs, and thus that the unclassified variants analysed in this paper do not have any effect on splicing. This would suggest that, despite efforts to reduce the level of false positives identified by current bioinformatic prediction programs [8,10], the accuracy of these programs has yet to approach the levels necessary for application in clinical scenarios. It has certainly been recognised that attempts to predict the site of ESEs are often confounded not only by the high level of variability accommodated by motifs [7], but also because ESE function is defined by additional factors, such as the distance from the splice site and splice site strength [20]. Consistent with this, different prediction programs regularly produce conflicting results [21]. The incorporation of evolutionary conservation filtering on ESE prediction improved the colocalization with reported unclassified variants [10] however, this may in fact reflect the pressure to maintain amino acid sequence rather than an ESE sequence.

The results of this study are supported by other studies demonstrating the validity of minigene reporter assays for analysing pre-mRNA splicing associated with DNA sequence variants [13,14,21], particularly when patient peripheral blood or LCLs are unavailable for RNA analysis [19]. An additional advantage of minigene assays is that the effect on splicing produced by a variant allele can be isolated from the influence of a wild type allele.

## Conclusions

The increased reporting of rare sequence variants with routine sequencing of high-risk genes in *BRCA1 *and *BRCA2 *has resulted in increased reporting of unclassified variants, a situation that will be exacerbated for *all *disease-predisposition genes as deep sequencing becomes more affordable for clinical testing laboratories. Web-based splice prediction tools have an important role in assessing and prioritizing variants for analysis using experimental methods. Validating predictions using *in vitro *techniques will provide valuable data for the development of *in silico *tools, particularly those for ESEs where prediction accuracy is currently very poor [10,12,13,21]. Improving the predictive capabilities of web-based programs is essential before they can be utilised for large scale prioritisation of variants for assays, or for the long-term goal of prediction at the clinical level. Our results demonstrate the difficulty in predicting the impact on splicing of sequence variation within putative ESEs, and stress the importance of experimental validation of bioinformatic predictions.

## Abbreviations

ESE: Exonic splice enhancer; BIC: Breast Cancer Information Core; kConFab: Kathleen Cuningham Foundation Consortium for Research into Familial Breast Cancer; SR: Serine/Arginine rich.

## Competing interests

The authors declare that they have no competing interests.

## Authors' contributions

PJW carried out minigene assays and drafted and assisted in the revision of the manuscript. CAP performed RT-PCR analysis for one variant, preliminary bioinformatic analysis and contributed to manuscript preparation. LCW and BLB assisted with cell culture, molecular techniques and manuscript preparation. ABS assisted with manuscript preparation and interpretation. MAB designed and coordinated the study, and drafted and revised the manuscript. All authors have read and approved the final manuscript.

## Pre-publication history

The pre-publication history for this paper can be accessed here:

http://www.biomedcentral.com/1471-2350/11/80/prepub
